# Structural studies of CNG repeats

**DOI:** 10.1093/nar/gku536

**Published:** 2014-06-17

**Authors:** Agnieszka Kiliszek, Wojciech Rypniewski

**Affiliations:** Institute of Bioorganic Chemistry, Polish Academy of Sciences, Noskowskiego 12/14, 61–704 Poznan, Poland

## Abstract

CNG repeats (where N denotes one of the four natural nucleotides) are abundant in the human genome. Their tendency to undergo expansion can lead to hereditary diseases known as TREDs (trinucleotide repeat expansion disorders). The toxic factor can be protein, if the abnormal gene is expressed, or the gene transcript, or both. The gene transcripts have attracted much attention in the biomedical community, but their molecular structures have only recently been investigated. Model RNA molecules comprising CNG repeats fold into long hairpins whose stems generally conform to an A-type helix, in which the non-canonical N-N pairs are flanked by C-G and G-C pairs. Each homobasic pair is accommodated in the helical context in a unique manner, with consequences for the local helical parameters, solvent structure, electrostatic potential and potential to interact with ligands. The detailed three-dimensional profiles of RNA CNG repeats can be used in screening of compound libraries for potential therapeutics and in structure-based drug design. Here is a brief survey of the CNG structures published to date.

## INTRODUCTION

Trinucleotide repeats (TNRs) are a class of microsatellite sequences abundant in the intergenic as well as genetic regions, including open reading frames. More than 30 000 TNRs (six repeated units or more) have been found in the human genome (1). Similar to other microsatellites, TNR sequences exhibit high variability in length between individuals. The mutability of length and its overrepresentation in genes suggest that these can be regulatory elements or a source of evolutionary change, perhaps fine-tuning gene expressions (2). These TNR features can be functional in a normal organism, but they can become deleterious when abnormal lengthening of repeat units occurs. This is observed in humans with neurological diseases known as TREDs (trinucleotide repeat expansion disorders). One major subset of pathogenic TNRs is the CNG repeats, where N represents one of the four natural nucleotides. CNG repeats are associated with at least 15 diseases, such as myotonic dystrophy (DM), Huntington's disease, several spinocerebellar ataxias (SCA) and fragile X mental retardation syndrome (FXS) (3).

The current models of the pathomechanism of TREDs postulate two toxic agents: RNA and protein (4); however, recent reports also speculate about DNA toxicity (see reviews (2,5)). The RNA-mediated mechanism is based on the observation that long CNG repeats are transcribed and included in mRNA. There, they form hairpin structures that exhibit a gain-of-function abnormality by sequestering and accumulating regulatory proteins. This upsets the fine balance of cellular processes (6–8) and results in retention of the RNA and proteins in the nucleus, where they form nuclear foci (see review (9)). The second pathomechanism mostly concerns the toxicity of protein. The mutated protein contains elongated polyglutamine (polyQ) tracts, which result in the misfolding and aggregation of the protein (10). Most polyQ-containing proteins are involved in DNA-dependent regulation of transcription or neurogenesis. Moreover, they participate in multiple intermolecular contacts. Similar to the RNA-mediated mechanism, the polyQ-expanded region induces pathogenic interactions with proteins, leading to the formation of toxic mono- and oligomers. Subsequently, amyloid-like inclusions are formed, sequestering all engaged proteins (9).

In recent reviews, the sharp division of pathomechanisms started to blur and became more complex. In addition to the main mechanism of the development of some TREDs, another parallel toxic path has been suggested to coexist (see reviews (11–13)). This path is associated with bidirectional transcription, which normally results in antisense RNA involved in the regulation of gene expression. In cells affected with TREDs, the antisense transcripts can contain an extended run of CNG repeats complementary to the sense strand. Subsequently, the antisense RNA can undergo repeat-associated non-ATG (RAN) translation that occurs independently of an ATG initiation codon (14–16). The small expanded peptides can exhibit toxicity, similar to polyQ diseases. Another possibility is that antisense RNA can hybridize to sense RNA and form double-stranded structures, which can be processed into siRNA, derived from triplet repeats, activating the silencing mechanism (17,18). We cannot exclude that such RNAi is also generated from the hairpins formed by sense CNG transcripts (19–21). Bidirectional transcription is also associated with a path of DNA toxicity, and the convergent transcription from both strands triggers cell death (22). The double-bubble, which is formed at the expanded repeats by the collision of the sense and antisense transcription, is necessary for the induction of apoptosis (2).

To summarize, in recent years the pathomechanism of TREDs has been intensely investigated. A range of molecular biology techniques was used in *in vitro* and *ex vivo* systems and in *in vivo* models. Additionally, crystallography made a contribution to the study of TREDs. A number of structures have been reported in the last 10 years, mostly concerning RNA-mediated pathogenesis, and these findings have not yet been summarized. In this review, we present crystallographic models of toxic RNA and give an overall view of the structural features of the CNG repeats. We begin with a short description of the diseases associated with each type of repeat, followed by an introduction into the structural studies, including a short characterisation of the secondary structure of the DNA and/or RNA. Next, all crystallographic reports of RNA-mediated molecules are presented and discussed, which is followed by a description of the NMR and thermodynamic studies.

## TOXICITY OF EXPANDED CNG REPEATS

Abnormally expanded CUG repeats are best known for the multiple system dysfunctions that they cause in myotonic dystrophy type 1 (DM1) (23). The mutation leading to DM1 is the expansion of a CTG repeat in the 3′-untranslated region (3′-UTR) of the *dystrophia myotonica* protein kinase (*DMPK*) gene. The normal length of 5–37 CTG repeats is expanded in DM1 to 50–3000 repeats (24) and entails a misregulation of alternative splicing of several developmentally regulated transcripts (25,26). This misregulation is caused by altered interactions of the transcripts with two antagonistic splicing regulators: the CUG repeat binding protein (CUG-BP) (27) and the muscleblind-like (MBNL1) protein (28). The level of MBNL1 decreases as it is sequestered to nuclear foci (29,30), while the level of CUG-BP increases (31).

Elongated CAG repeats are best known to cause the poly-Q diseases, in which toxicity is attributed to malformed proteins derived from the affected genes (32,33). However, recent studies indicate that mutant transcripts can also contribute to pathogenesis (34,35). CAG repeats in transcripts are similar to CUG repeats in their interaction with the splicing regulator MBNL1 (34,36), whose sequestration by CUG tracts causes splicing aberrations leading to DM1 (28,37) and spinocerebellar ataxia type 8 (SCA8) (38). CAG repeats trigger neurodegeneration when introduced into a *Drosophila* SCA3 model (39). In all, expanded CAG repeats are the cause of nine human neurodegenerative disorders, including Huntington's disease and several spinocerebellar ataxias (40).

Expansion of CGG repeats in the 5′-untranslated region (5′-UTR) of the fragile X mental retardation gene (*FMR1*) is associated with several phenotypes of increasingly severe pathology, depending on the extent of elongation (41). The normal range found in the population is 5–54 CGG repeats (41–43), where the upper region of 45–54, defined as the ‘grey zone’, carries an increased likelihood of pathogenic expansion in descendants (41,44). Tracts of 55–200 CGGs are premutations and can cause a progressive RNA-mediated neurodegenerative disorder known as fragile X-associated tremor ataxia syndrome (FXTAS) (45,46) in elderly males, while females carrying the permutation are at risk of developing premature ovarian failure (47). More than 200 CGG repeats are full mutations resulting in fragile X syndrome (FXS), the most common inherited mental retardation syndrome in man (48).

CCG repeats are highly overexpressed in exons of the human genome and are typically located in the 5′-UTR or in the translated regions (1). Their role in pathogenesis is relatively obscure compared to the other CNG repeats, but they are found to be associated with three tri-nucleotide disorders: Huntington's disease (49), myotonic dystrophy type 1 (50) and chromosome X-linked mental retardation (FRAXE) (51).

## SECONDARY STRUCTURE OF CNG REPEATS

The secondary structure of RNA containing CNG repeats has been investigated extensively using *in vitro* models (for more detail, see reviews (52,53)). The models studied included RNA comprising not only CNG repeats but also transcripts of native mRNA. Digestion by nuclease S1 and ribonucleases T1, T2 and V1 as well as lead cleavage of oligomers comprising 17 CNG repeats indicated that all CNG repeats formed hairpin structures and that these hairpins showed several alternative alignments co-existing under non-denaturing polyacrylamide gel electrophoresis conditions (54). Adding terminal C-G ‘clamps’ reduced this micro-heterogeneity. CNG hairpins clamped by six C-G pairs showed only one alignment. The hairpins, comprising an odd number of CNG repeats, formed apical loops of four nucleotide (nt) residues, as indicated by susceptibility to nuclease digestion. In the case of odd-numbered repeats, CAG and CCG showed 7-nt loops, while CUG and CGG formed tighter 3-nt loops.

Further biophysical and biochemical studies to ascertain the structural diversity of a wider range of RNA triplet repeats categorized all CNG repeats as ‘fairly stable hairpins’, of which CGG was the most stable and CCG the least stable (55). Addressing the question of the possibility of CGG repeats forming quadruplexes, the authors found no evidence for their formation and concluded that the properties of CGG repeats did not essentially deviate from those of CUG, CCG and CUG repeats.

A more focussed study was carried out on CAG repeats in transcripts related to human diseases: the spinocerebellar ataxia types 3 and 6 and dentatorubral-pallidoluysian atrophy. The cleavage patterns of the transcripts, obtained by digestion with lead and a variety of ribonucleases, indicated the formation of several variants of a slipped hairpin (56). The study also demonstrated that a single-nucleotide polymorphism found near the CAG repeat modulated the structure. A related paper addressed the effect of naturally occurring 'interruptions' in an expanded CAG repeat in the coding sequence of the spinocerebellar ataxia type 1 (57). The cleavage patterns indicated that the interruptions destabilized the hairpin structure, causing specific bulging and branching. This was proposed to mitigate the onset of pathogenesis. A similar investigation of CAG-repeat-containing transcripts related to spinocerebellar ataxia type 2 revealed the structure-modulating role of naturally occurring CAA interruptions (58). Single-nucleotide polymorphism was also observed in the CGG repeats in FMR1 gene transcripts. Single AGG interruptions changed the folding of the 5′-UTR fragments, resulting in branched hairpin structures (43).

DNA containing an extended number of CNG repeats forms non-B DNA structures (59), which can occur during replication, translation, recombination and repair. During those processes, the two parental strands are separated and single-stranded DNA structures can be formed. The secondary structure of isolated dCNG repeats has been studied by a variety of methods, such as electrophoretic mobility assay, UV absorbance and chemical or enzymatic digestion (see review (60). Similar to RNA, all four types of isolated repeats form hairpin structures. The G-rich dCGG repeats also have the potential to form tetraplexes. In the presence of K^+^ ions, the d(CGG)_20_ oligomer exhibited increased electrophoretic mobility, suggesting that it formed an intramolecular tetraplex (a hairpin folded in half). This result was confirmed by CD, NMR and UV spectroscopy (59).

To date, there is only one direct line of evidence showing that the DNA hairpin structures are formed *in vivo*. Two distinct zinc finger nucleases (ZNF) were engineered to recognize specifically the stem of a hairpin formed by CAG or CTG repeats (61). The nucleases were expressed in cells containing an extended number of repeats. As a result, a contraction of the repeated sequence was observed, indicating the presence of hairpins. Moreover, nuclease activity was detected only in an active replication state and only in cells harbouring 45 or 102 repeated units.

## X-RAY CRYSTALLOGRAPHIC STUDIES

Crystal structures have been published of RNA oligomers containing all four types of CNG repeats.

### CUG repeats

**Figure 1.**
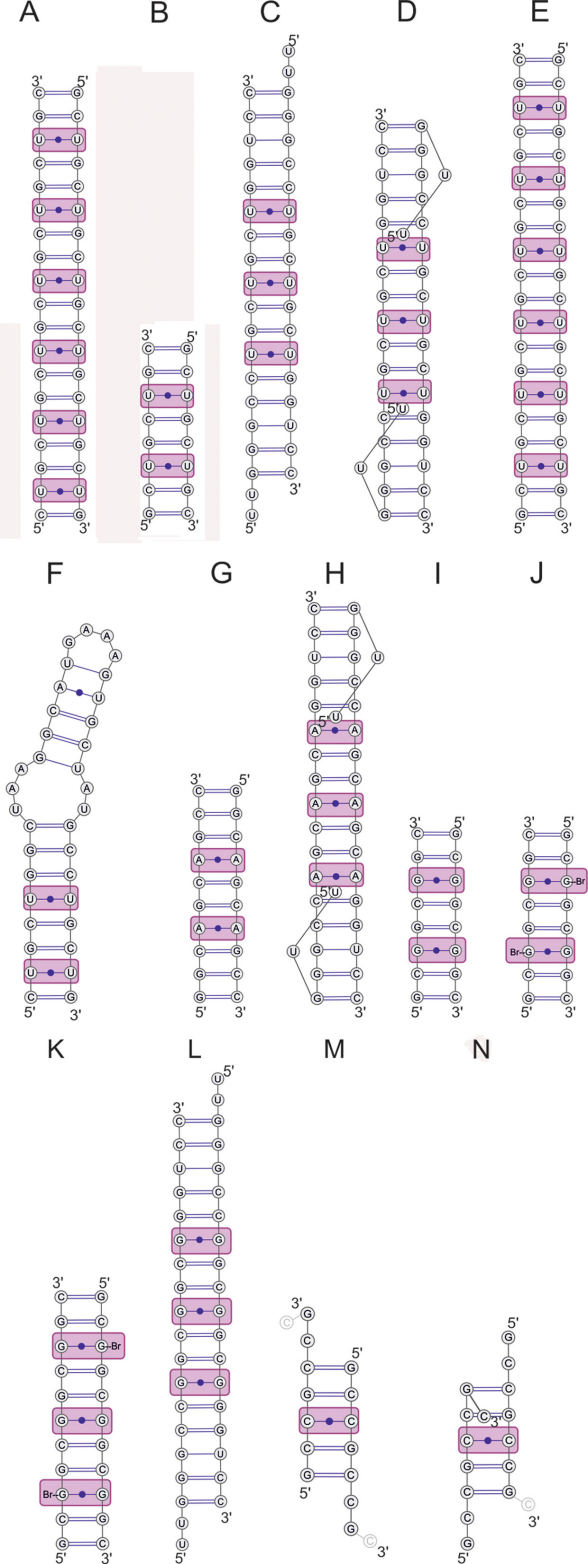
Base-pairing diagrams of the CNG-containing oligomers, whose crystallographic structures have been solved. Structures containing CUG repeats (**A–F**), CAG (**G, H**), CGG (**I-L**) and CCG (**M, N**) are described in the text.

**Figure 2.**
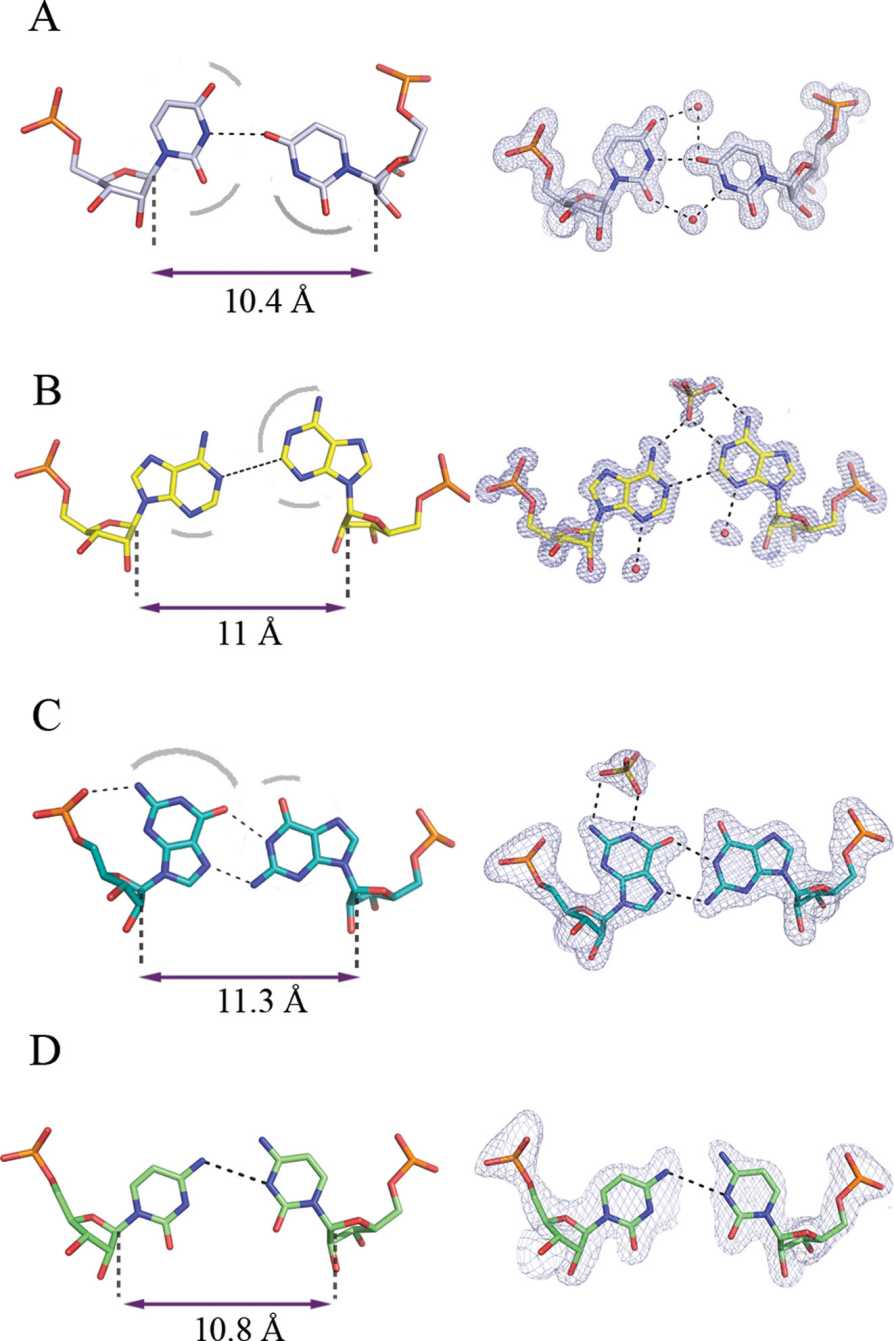
Non-canonical N-N pairs within duplexes formed by CNG repeats: U-U (**A**), A-A (**B**), G-G (**C**) and C-C (**D**). Strand separation is indicated, measured as a distance between the C1’ atoms. Surfaces especially liable to interact with the solvent through H-bonding are indicated with arcs. Representative N-N pairs with the *2Fo-Fc* electron density, contoured at 1σ level, are shown (right) based on PDB entries 3g1p, 3nj6, 3r1c, 4e59, respectively.

The earliest crystallographic study of a trinucleotide repeat targeted the structure of an oligomer comprising six CUG repeats (PDB code 1zev) (62). The authors found that the RNA is double-helical, having overall characteristics of the A-form, in which the C-G and G-C base pairs have U-U ‘mismatches’ in between (Figure 1A). The non-canonical pairing seemed not to distort the backbone from the A-helix. Closer observations of the inter-strand interactions, in particular the details of the non-canonical base-pairing and the solvent structure, were prevented by an apparent superposition of molecules in the crystal lattice. A different study revealed duplexes of G(CUG)_2_C at a resolution of 1.23 Å (Figure 1B, PDB code 3g1p) (63). This RNA was also in the A-form with the helical twist in the typical range of 32–34°. The crystal lattice comprised three duplexes, crystallographically independent but with similar structures, stacked end-to-end to form pseudo-infinite helices. This arrangement is frequently found in crystals of nucleic acids. The most notable features of this structure are the clearly resolved interactions within the duplex and the well-defined solvent structure. While the C-G and G-C pairs formed standard Watson-Crick pairing, the U-U pairs interacted in a unique way. The chemical symmetry of the U-U pair is broken in the three-dimensional structure, which has one of the uridines inclined toward the minor groove to make a single hydrogen bond between its carbonyl O4 atom and the N3 amino group of the opposite base (Figure 2A). This interaction appeared ‘stretched’ compared to the consensus structure derived from previously observed U-U pairs (64) that had two hydrogen bonds. This stretching is clear when the C1’-C1’ distance between the two opposite uridine residues is considered. It amounts to 10.4 Å in the context of the CUG repeat, compared to the general consensus of 8.6 Å. A search using FRABASE (65) and FR3D (66) showed stretched U-U pairs in only two other structures: tRNA-Gln, in which the U-U pair closes the anticodon loop, and the A site of 16S rRNA, in which the helix is flanked by a G-C pair at the 5′ side and a C-G pair at the 3′ side.

**Figure 3.**
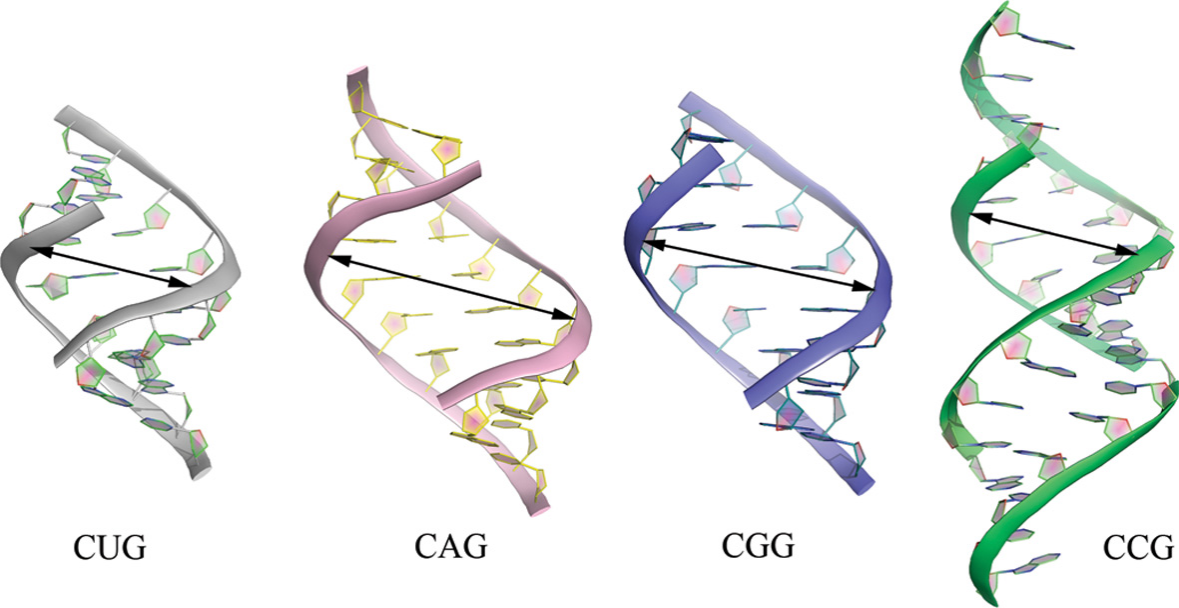
Duplexes containing CNG pairs. Local unwinding and subsequent widening of the major groove can be seen in the vicinity of A-A and G-G pairs.

**Figure 4.**
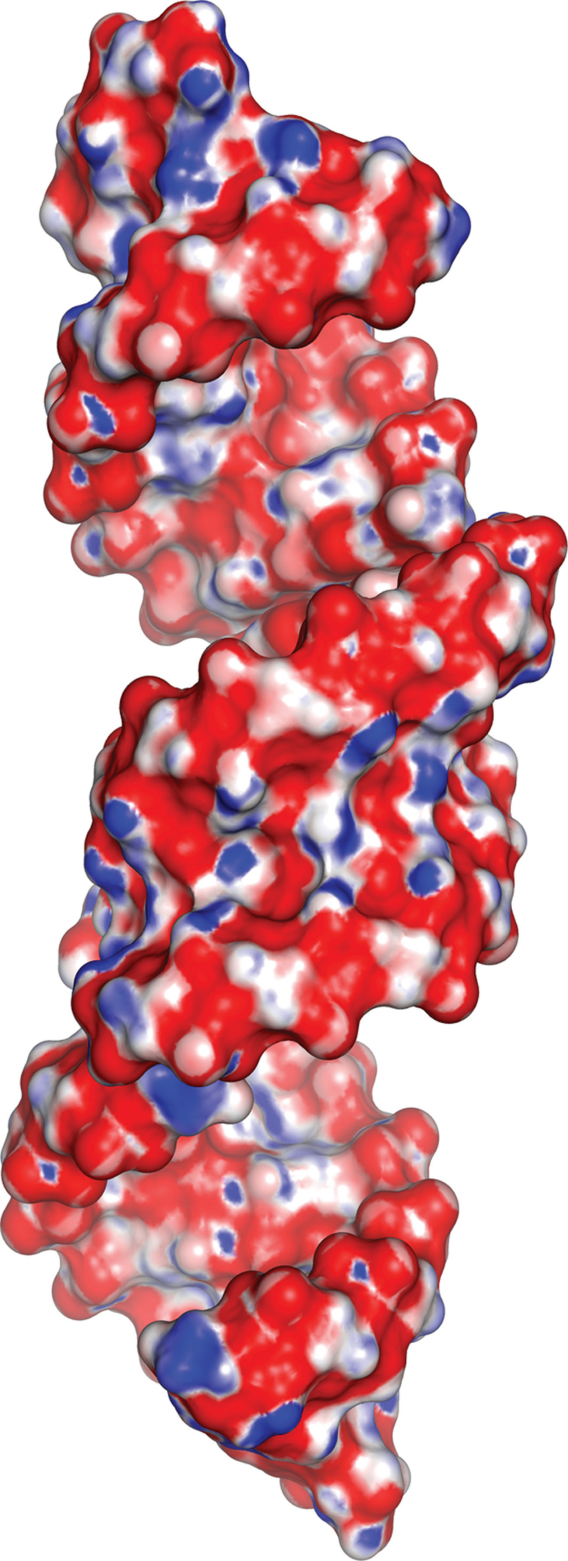
Electrostatic surface potential of three consecutive (GCUGCUGC)_2_ duplexes. Red is negative, blue is positive. The minor groove shows a striped pattern of surface potential characteristic of CNG repeats. Based on PDB entry 3gm7.

What keeps the uridines apart and prevents them from realising their full base-pairing potential? It is likely that this non-canonical pairing is stabilized by the strong flanking C-G and G-C pairs, which maintain the duplex in a clearly recognisable A-form (Figure 3A). The stability of the U-U pairing is reinforced by ordered water molecules in the major and minor grooves. These waters contribute to the H-bonding network and can be considered a part of the structure. Duplexes comprising CUG repeats have a characteristic pattern of surface electrostatic potential in their minor groove (Figure 4) that comprises alternating bands of positive and negative potential along the direction of the helix axis. The major groove shows no regularity and is predominantly electronegative with positive patches. The hydrogen-bonding potential of the duplexes is demonstrated by their interactions with ordered water molecules and small ligands present in the crystallisation medium (glycerol and sulphate ions). These interactions could be used as a guide in structure-based drug design. The paper also includes a new analysis of the data obtained by Mooers *et al.* (62). After detwinning the diffraction intensities, the structure of the [(CUG)_6_]_2_ duplex (PDB code 3gm7) corresponds closely with the structure of the shorter duplexes.

Given that each U-U pair has two possible conformations, depending on which uridine is inclined, it begs the question: is the choice random or is there a correlation between the conformations of adjacent pairs? The structures included in this study indicate that there is no correlation. This means that despite the chemical symmetry and the palindromic nature of duplexes comprising CUG repeats, the number of possible conformations is large and grows rapidly for longer runs: approximately as 2^N^/2 for N repeats. The authors suggested this as a way of explaining how long runs of CUG repeats could differ from short runs and condense into nuclear foci, as opposed to shorter repeat sequences that remain soluble.

Another study of a crystal structure containing CUG repeats reported a wider range of conformations of the U-U pairs, including arrangements with zero, one or two H-bonds (67). Two crystal forms were reported of a double-stranded construct containing three CUG repeats and 5′UU dangling ends (Figure 1C, D; PDB codes 3szx, 3syw). In both crystal forms, the central CUGs had the uracil rings symmetrically opposed and apparently too far apart to form any H-bonds. In the flanking repeats, the two structures differed. In one of them, the first U-U pair formed a single O4-N3 H-bond, while the other pair made a tenuous 3.6 Å contact. The distance between the RNA strands was at least 10 Å and the dangling ends interacted with neighbouring duplexes to form a crystal lattice of pseudo-infinite helices. In the other crystal form, the dangling ends were tucked in the major groove and the width of the groove was increased, while the distance between the strands decreased below 10 Å, which allowed the interacting uridines to form two hydrogen bonds. Based on the structural variation in the crystal, the authors postulated that the U-U pairs could sample multiple conformations *in vivo*, which has implications for the recognition by proteins and small ligands.

A crystal structure of G(CUG)_6_C 20-mers forming blunt-ended double helices was described recently (Figure 1E) (68). The crystal lattice contained two distinct duplexes of which one was located on a crystallographic 2-fold axis (PDB code 4e48). Thus, the structure contained nine distinct double-helical CUG repeats. The authors took the opportunity to compare the nine U-U pairs, analyse the spread of conformations and thus gain insight into the dynamics of such pairing. They found that the ‘stretched U-U wobble’ with a single H-bond and associated semi-conserved water structure was the predominant mode of U-U pairing (7 instances), but they also found interesting deviations. One U-U pair exhibited a clearly symmetric pairing mode, described as ‘symmetric H-nonbonded U-U pairing’, which the authors proposed as an intermediate conformation between two predominant asymmetric pairings. The different modes of interactions displayed by the U-U pairs could result in the adaptability of CUG repeats in the protein-bound state. The authors concluded that ‘generally speaking, the U-U pairs just follow the requirements of the system’.

The most recent report regarding the CUG series presents the crystal structure of an RNA construct containing a tandem GAAA tetraloop and its receptor that form the tip and the upper part of a hairpin, respectively, while the lower part of the stem is a double-helical segment of two CUG repeats (Figure 1F, PDB code 4fnj) (69). The combination of the tetraloop with the receptor facilitated crystallisation, which enabled the examination of yet another instance of two consecutive CUG repeats. They formed a helix whose geometry also corresponded to the A-form. The C-G and G-C pairs were standard Watson-Crick pairs, while the two U-U pairs displayed two of the previously observed conformations: one with one hydrogen bond and one with two. The authors catalogued all U-U conformations observed to date and observed that the most common U-U pairing in the context of CUG repeats was the one with one H-bond and a strand separation of at least 10 Å, consistent with the geometry of the A-form. The authors concluded that ‘U-U pairs are dynamic’. This is an understandable statement, but it requires some caution. One could postulate that the multiple conformations are ‘snapshots’ of a dynamic system, but in crystallography, we do not actually observe the transitions between the different instances and therefore cannot be clear about their nature—in particular, their frequency and the conditions upon which they depend.

### CAG repeats

Two crystal forms of (GGCAGCAGCC)_2_ have been reported (70). One of the structures was solved at atomic resolution (0.95 Å) and contained a duplex with the two strands related by crystallographic symmetry (Figure 1G, PDB code 3nj6). The other structure was analysed at medium resolution (1.9 Å) and contained three crystallographically independent duplexes (PDB code 3nj7). All duplexes were closely superposable and possessed general characteristics of the A-form. The A-A pairs, embedded between the canonical C-G and G-C pairs, had both residues in the *anti* conformation (Figure 2B). Clashing between the large purine rings was avoided by shifting of the residues towards the major groove. One was shifted more than the other and this was described as the 'thumbs-up' conformation. The mutual positioning of the adenines allowed one weak C2H2···N1 hydrogen bond. To our knowledge, such pairing between adenosine residues had not been described. The non-canonical character of the interaction resulted in local distortions of the helix geometry – in particular, in the flipping of the O5’ atom of the backbone of the neighbouring guanosine, due to a rotation of the C5’-O5’ bond. This resulted in a local unwinding of the helix. Although accommodation of the bulky adenine rings within the helical context seems to be 'sterically demanding', the inter-strand C1’-C1’ distances (11 Å) for the adenosines were only slightly larger than average for the A-form. The unsaturated H-bonding potential of the A-A pair (both *exo*-amino groups and one N1 atom) was externalized in the major groove where it attracted a sulphate anion from the crystallisation medium. Patches of positive potential on the predominantly negative interior of the major groove were clearly visible on the surface electrostatic potential. The minor groove displayed a similar banded pattern of alternating positive and negative potential, similar to that seen in CUG repeats.

In another study, three consecutive CAG repeats within flanking sequences were described (Figure 1H) (71). The middle A-A pair had both adenosines in the *anti* conformation and the two adenine rings were modelled as being symmetrically opposed with the N1 atoms and the C2H2 groups in unlikely close contact. Examination of the electron density (see Appendix), calculated on the basis of structure factors deposited by the authors in the PDB (accession code 4j50), indicates disorder, which could be modelled as two alternative conformations of the A-A pair. In each of the conformations, one adenine would be at more of an incline than the other, so that C2H2 would be opposite N1 and a hydrogen bond could form between them, similar to the structures reported before (70). Uninterpreted electron density in the major groove near the A-A pair could indicate a sulphate ion interacting with the *exo*-amino groups, like in the previous paper. The flanking A-A pairs were described as *syn-anti*. The adenine in the *anti* conformation has clear electron density, while the residue in the *syn* conformation appears to be rather disordered. There are significant residual peaks in the difference electron density map associated with the adenine ring, and the sugar-phosphate backbone is weak in this region. It is not clear how this should be modelled. The adenosine in the *syn* conformation seems to interact with the overhanging disordered uridine from the opposite strand that is tucked in the major groove.

### CGG repeats

Three crystal structures of short CGG-containing oligomers have been published (Figure 1I-K) (72). A duplex of G(CGG)_2_C crystallized with a remarkable 18 distinct duplexes in the unit cell, all arranged in the typical end-to-end manner, in semi-infinite columns (PDB code 3r1c). The other two crystal structures contained guanosine residues brominated at position 8 (PDB codes 3r1d and 3r1e). All helices had the A-form, with some local deviations. In all G-G pairs, one guanosine was *syn* and the other was *anti*, with two hydrogen bonds between the Watson-Crick edge of G(*anti*) and the Hoogsteen edge of G(*syn*): O6···N1H and N7···N2H (Figure 2C). This type of interaction is common for G-G pairs and has been observed in many NMR and crystallographic structures. The G(*syn*) residues also showed unusual α and γ backbone torsion angles, which resulted in a local unwinding of the helix, which seemed to be compensated for elsewhere (Figure 3C). The G(*syn*)-G(*anti*) pairs had a characteristic hydration pattern and, in addition, attracted charged species from the solvent, especially to the exposed Watson-Crick edges in the major groove. Sulphate anions and hydrated calcium ions, present in the crystallisation medium, interacted with the paired residues. The *syn-anti* arrangement avoids a steric clash of the two bulky guanines within the helical structure, and the C1’-C1’ distance between them (11.3 Å on average) is only slightly longer than that for the canonical C-G and G-C flanking pairs. The brominated guanosines were always in the *syn* conformation, which increased the order of the G-G pair by restricting its conformational freedom. The electrostatic potential surface displayed the already familiar banded pattern of positive and negative character in the minor groove. This is due primarily to the C-G and G-C pairs. The potential in the major groove was irregular and matched the observed affinity for small charged ligands. The observation of duplexes for all CGG-containing structures was somewhat surprising to some researchers, who expected quadruplexes by analogy with DNA.

Another paper described an RNA duplex containing three consecutive CGG repeats with flanking sequences and 5′-UU overhangs (Figure 1L) (73). Overall, the structure of the CGG repeats is similar to that described above (72), with G(*syn*)-G(*anti*) pairs and unusual torsion angles of the G(*syn*) residues, resulting in a local unwinding of the helix (PDB code 3js2). The authors noted that the resultant widening of the major groove and the base-pair inclination near the G-G pairs resembled the A′-form of RNA. They also noted an interesting difference from the structures of other triplet repeats: the lack of ordered ions near the G-G pairs, even though several cations and sulphate anions were present in the crystallisation medium.

### CCG repeats

At present, there is one paper reporting the crystallographic structure of CCG repeats (74). It describes two oligomers: (GCCGCCGC)_2_ and (GCCG^L^CCGC)_2_, in which G^L^ belongs to the ‘locked’ (LNA) series (Figure 1M, N). The oligomers formed duplexes in the crystal lattice, and again, the RNA has the A-form, but pairing of the strands was unexpected. In the unmodified oligomer, the strands slipped in the 5′ direction (PDB code 4e59), whereas in the LNA-containing oligomer, there was a slippage in the 3′ direction (PDB code 4e58). In both cases, the result was to reduce the number of C-C pairs from the expected two, if no slippage had occurred, to one. Nevertheless, three instances of double-stranded CCG triplets were observed: one for unmodified RNA and two in crystallographically independent LNA-containing duplexes. Each of the three observed C-C pairs interacts differently, forming either one weak H-bond or none (Figure 2D). LNA has no apparent effect on helical parameters, but base stacking is increased compared to the native duplex. It seems that C-C pairs contribute little to the stability of the duplex, which is why the system acts to eliminate them by strand slippage. These results are in agreement with the measured thermodynamic fragility of CCG repeats. C-C pairs within other known helical RNA structures are relatively rare, but if present, they also show conformational variability. One of the cytosine residues is shifted to various extents towards the minor groove and different H-bonds are observed between the paired cytosines. The apparent weakness of the C-C interactions also sheds light on the observation that the MBNL1 protein, thought to interact with single strands of RNA, recognizes CCG runs as well as CUG and CAG but not the relatively robustly paired CGG repeats.

## NMR STUDIES

There are relatively few published works describing NMR studies of RNA TNRs.

The earliest paper presents an analysis of a 97-long run of CUG repeats in solid state (75). The authors noted the presence of canonical C-G pairs and observed resonances consistent with an A-form helix with a C3′-*endo* sugar pucker and an *anti* conformation of the glycosidic torsion angle. Recent solution NMR work on a single CUG with flanking sequences, to stabilize the duplex form, was also consistent with an A-form geometry with a 3C′-*endo* sugar pucker (PDB codes 2l8c, 2l8u and 2l8w) (76). The line broadening and temperature profile of the spectrum indicated a structurally dynamic U-U pair. When the NMR model was subjected to molecular dynamics simulation, the U-U pair was found to adopt conformations with zero, one or two hydrogen bonds, of which the most stable was the structure with one H-bond. These results are essentially in agreement with the crystallographic studies described above.

One paper describes a study of CGG-repeat RNA in solution (77). One short duplex and two hairpins, each predicted to contain three CGG repeats, were investigated. The duplex gave an ambiguous spectrum and most likely formed longer than predicted, overlapping duplexes. The authors observed no patterns characteristic of quadruplexes. The oligomers designed to fold into hairpins gave spectra indicating that hairpins indeed formed and that the non-canonical G-G pairs were located between flanking C-G and G-C pairs. The G-G pairs appeared dynamic with some indication of symmetric G-imino–G-imino interactions, but glycosidic bond angles necessary for such an interaction could not be determined due to severe line broadening and signal overlap. The authors again stressed that there was, nevertheless, no evidence of tetraplex formation.

**Figure 5.**
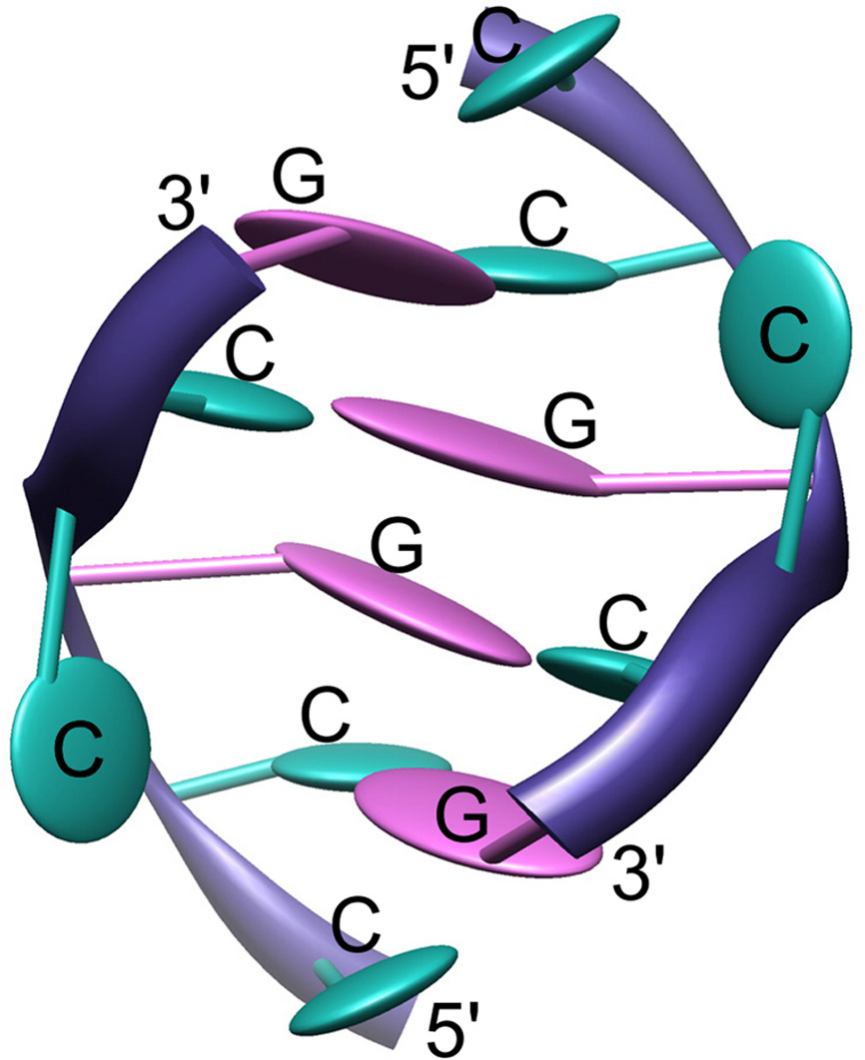
No C-C pairs are formed in the solution NMR structure of (CCGCCG)_2_ DNA because the cytosine residues hang out or bulge out of the double helix.

Only one three-dimensional structure has been published of DNA containing CNG repeats (78). The solution structure of d(CCGCCG)_2_ was solved using NMR (PDB code 1noq). The double helix contained only G-C and C-G pairs. The C-C pairs were eliminated by strand slippage, which resulted in dangling cytosine residues at the 5′ site of each strand. In addition, both 4C residues bulged out causing deformation of the phosphate backbone (Figure 5). The structure shows similarity with the crystallographic models in that the unstable C-C pairs have been eliminated.

## THERMODYNAMIC PROPERTIES OF CNG REPEATS VERSUS CRYSTALLOGRAPHIC DATA

Thermodynamic studies have shown that RNA oligomers containing 2–3 repeats form duplexes, while oligomers with 4–5 repeated units exist as a mixture of duplexes and hairpins. Longer RNA molecules form only hairpins (79). Duplexes of G(CNG)_2–4_C oligomers have comparable thermodynamic stability. Oligomers of CGG repeats have the lowest free energy (ΔG_37°_) (approx. −10 kcal/mol) followed by comparable oligomers of CUG repeats (−7.5 kcal/mol), then CAG ≈ CCG (−6.5 kcal/mol). Hairpin structures containing 5–7 CNG units also have comparable stability, but the most stable repeats are CUG, followed by CAG, then CGG and CCG. However, such oligomers form a mixture of structures and deconvolution of the melting curves is necessary to obtain the thermodynamic parameters of each type of structure. The results for longer RNA molecules containing 20 repeats agree with those obtained for shorter oligomers (55). Values of ΔG_37°_ range from −2.38 to −6.68 kcal/mol. In 100 mM NaCl, the most thermodynamically stable were CGG, then CAG, CUG and CCG repeats. However, measurements performed in 100 mM KCl gave different results. The stability series began with CAG repeats followed by CGG, CUG and CCG. The biggest drop in thermal stability was observed for the CGG repeats. The destabilising effect on other TNR RNA was lower: in the range of 0.18–0.69 kcal/mol. The loss of stability in the presence of K^+^ ions was also an indication that none of the RNA formed tetraplexes, which are generally promoted by the presence of K^+^.

Thermodynamic stability is an important property of nucleic acids and is difficult to investigate using crystallography, although some estimations are possible based on the number of H-bonds in the N-N pairs or the area of stacking interactions. Considering the aforementioned parameters in the crystallographic structures of TNR RNA, the CGG repeats appear the most stable. The G-G pairs form the largest number of hydrogen bonds (in addition to two H-bonds between the paired guanines, the residue in the *syn* conformation bonds the phosphate group of its own strand). The CUG repeats form the second most stable structure due to the one regular H-bond within the U-U pair. The A-A pairs within the CAG repeats interact *via* one weak C-H···N hydrogen bond. In the case of the CCG repeats, the system limits the number of non-canonical pairs, which do not readily form H-bonds. However, the thermodynamic data for duplexes of (GCUGCUGC)_2_ and for (GCCGCCGC)_2_ show interesting properties. RNA containing CCG repeats is more stable (ΔG_37°_ = −6.09 kcal/mol) than CUG repeats (ΔG_37°_ = −5.08 kcal/mol). For longer oligomers (3 and 4 repeated units), the stability increases for CUG (ΔG_37°_ > 7 kcal/mol), while for CCG, it remains nearly the same. This effect is difficult to explain, but in the CUG repeats structure, this can be due to limited stacking interactions that increase with oligomer length. In the case of the CCG repeats, this suggests that strand slippage occurs as observed in the crystallographic models. One of the C-C pair is eliminated, giving energetic gain for the short RNA. For longer oligomers, the system is most likely not able to reduce the number of non-canonical pairs destabilising the RNA structure.

Interestingly, in our experience, the ease of crystallisation and the crystal reproducibility of the oligomer corresponded to the thermodynamic properties of the particular type of CNG repeat. The CGG oligomers crystallized rapidly, but it was difficult to obtain monocrystals. Most likely, the conformational flexibility of the G-G pair was introducing disorder that was overcome by seeding. In the case of CUG, good crystals appeared within several days. The oligomers of the CAG and CCG repeats were the most challenging for crystallisation. The crystal reproducibility was random and attempts to optimize crystallisation did not give satisfactory results. It seems that serendipitous changes in the fragile equilibrium in the crystallisation drops enabled us to obtain single crystals.

## CONCLUSIONS

All CNG repeats fold into hairpins in which the double-stranded stems have non-canonical N-N pairs that are stabilized by the sturdy C-G and G-C pairs. The A-form prevails albeit with some deviations characteristic of the given N-N pair. All CNG duplexes have been characterized in crystallographic detail in terms of their base-pairing, interactions with the solvent and small ligands, detailed helical parameters and deviations from the canonical A-helix as well as their electrostatic potential and potential to form hydrogen bonds. Their three-dimensional structural profiles are detailed enough to serve as a basis for screening compound libraries for potentially useful ligands and for structure-based drug design.

## PERSPECTIVES

Time has come to move on to more complex structures. To begin with, the picture of hairpins formed by CNG repeats is incomplete. Although detailed structures of their double-stranded stems have been solved, we still do not know the structure of the apical loops. Second, to understand the toxicity of the expanded CNG repeats, one would need to examine their interactions with other molecules and their role in altered gene splicing and the formation of nuclear foci. With this knowledge, one could search in a rational way for ligands that would bind the CNG runs to mitigate their deleterious effects.

This question is addressed in a paper reporting the crystallographic complex of zinc finger domains from the alternative splicing regulator protein MBNL1 and CGCUGU (80). The model shows the protein interacting with a short single-stranded RNA, in particular with the GC step in its sequence. Inspection of the atomic coordinates and the electron density calculated using the structure factors deposited with the PDB (code 3d2s) reveals unexplained degradation of the RNA. From a crystallographer's perspective, it reveals a puzzling, nearly perfect match between pairs of protein molecules and the associated RNA chains, which are related by a translation of half a unit cell along the crystallographic *a*-axis. Further studies are clearly necessary to elucidate the interactions of CNG repeats with their protein partners.

Another clear aim for further studies is a detailed characterisation of complexes between CNG repeats and compounds that could bind them specifically and, hopefully, reverse the precipitation of nuclear foci or prevent their formation. Several classes of compounds have been proposed as potential therapeutics (81–84). Examining their interactions with CNG runs would help verify their utility and enable their refinement.

## APPENDIX

Examining the atomic coordinates and the corresponding electron density is a useful addition to reading crystallographic papers, and it can be accomplished easily, even by non-crystallographers. Freely available applications such as Coot (www2.mrc-lmb.cam.ac.uk/Personal/pemsley/coot) automatically download atomic models and calculate electron density maps; one needs only to enter the PDB code to view this information.

## FUNDING

National Science Centre [Poland, UMO-2011/01/B/NZ1/04429]. Source of open access funding: National Science Centre [Poland, UMO-2011/01/B/NZ1/04429].

*Conflict of interest statement*. None declared.
